# A novel finding related to bulla and bleb formation in patients with primary spontaneous pneumothorax

**DOI:** 10.1186/s12890-021-01402-z

**Published:** 2021-01-09

**Authors:** Akira Iyoda, Yoko Azuma, Takashi Sakai, Satoshi Koezuka, Hajime Otsuka, Atsushi Sano

**Affiliations:** Division of Chest Surgery, Department of Surgery, Toho University School of Medicine, 6-11-1, Omori-Nishi, Ota-ku, Tokyo 143-8541 Japan

**Keywords:** Pneumothorax, Inflation, Airway

## Abstract

**Background:**

Spontaneous pneumothorax is a common problem globally. Bullas and blebs have been implicated in this problem, but the etiology of their formation is unknown. We aim to show the relation between a novel clinical finding, the pulmonary delayed inflation (PDI) sign, and the etiology of bulla and bleb formation in young patients.

**Methods:**

We retrospectively analyzed data from 111 patients with pneumothorax and a control group of 27 patients. We evaluated the relation between the PDI sign and other clinical factors.

**Results:**

The PDI sign was observed in 78 patients. Of these, 75 exhibited the PDI sign in only the upper lobe. Regardless of smoking status, patients 34 years of age or younger had a significantly higher incidence of the PDI sign than, patients 55 years of age or older and control patients. The inflation time in patients 34 years of age or younger, regardless of smoking status, was significantly longer than in patients 55 years of age or older and patients in the control group. There was no significant association between inflation time and the presence of asthma.

**Conclusions:**

The novel PDI sign is seen in patients 34 years of age or younger. Because this sign may indicate a peripheral bronchial abnormality and may be related to the formation of blebs and bullae in young patients with spontaneous pneumothorax, it is possible that it can be used to develop effective treatments for pneumothorax in young patients.

## Background

The incidence of spontaneous pneumothorax is 17 to 24 in 100,000 for men and one to six in 100,000 for women [1–3]. Although it is a common problem globally, the best method of management remains controversial [4]. One reason for this lack of consensus is that there are many categories of spontaneous pneumothorax. Primary spontaneous pneumothorax occurs in patients without underlying clinical lung disease, and secondary spontaneous pneumothorax is caused by underlying lung disease [5]. There is a bimodal age distribution to spontaneous pneumothorax, with one peak occurring in patients 55 years of age or older. These patients typically have secondary spontaneous pneumothorax, mainly caused by chronic obstructive pulmonary disease (COPD) [4]. The other peak is seen in young patients [1–3] and is related to male sex, taller height, and the formation of bullas or blebs [5]. In patients with secondary spontaneous pneumothorax, the fundamental treatment is smoking cessation. The fundamental treatment of primary spontaneous pneumothorax in young nonsmokers is unknown. Also unknown is the reason why bullas and blebs are formed in the apex of the lung [6] and how to prevent this formation. Many researchers have tried to discover the answer, without success. Tall height is frequently referred to as a risk factor for primary spontaneous pneumothorax: the hypothetical mechanism herein is that increased distending pressures on the apex of the lung are caused by the increased pressure gradient from the base to the apex of the lung in tall patients [5]. However, as imaging techniques have developed, bullas and blebs have come to be seen as emphysematous-like changes, and the concept that only ruptured bullas and blebs cause primary spontaneous pneumothorax is now obsolete [5]. Primary spontaneous pneumothorax may be related to a parenchymal abnormality, [7] or the primary pathologic process may involve inflammatory cells obstructing small airways and bronchiolitis [8]. These theories, based on pathologic findings, are currently under investigation. If these are determined to cause spontaneous pneumothorax, treating these factors will become the primary treatment for spontaneous pneumothorax.

We have noted a novel intraoperative finding that may explain why pleural changes, including bullas and blebs, are frequently observed in the upper lobe of the lung, and why those pleural changes are associated with abnormalities of the peripheral airways. We aim to show the relation between this novel finding, the pulmonary delayed inflation (PDI) sign, and spontaneous pneumothorax in young patients.

## Methods

This study was approved by the Ethics Committee of Toho University Omori Medical Center (M20033).

**Fig. 1:**
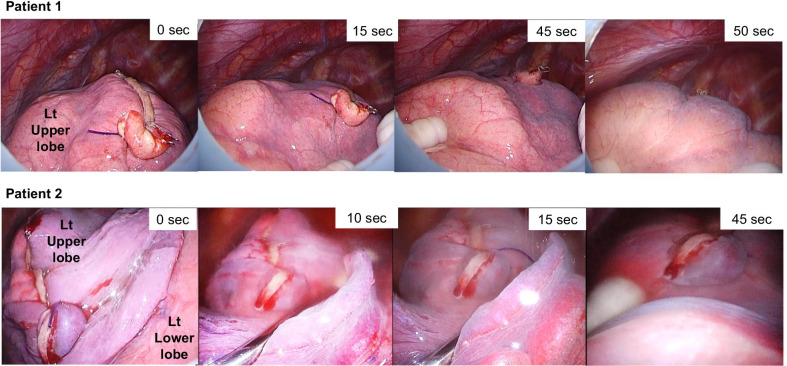
In patient 1, the left lower lobe is inflated, but the upper lobe is not. A total of 50 s were required to inflate the left upper lobe with an airway pressure of 20 cm H_2_O. In patient 2, the left upper lobe is collapsed despite adequate inflation of the lower lobe. With an airway pressure of 20 cm H_2_O, the upper lobe underwent gradual inflation.

We retrospectively analyzed data from all patients with pneumothorax and thoracoscopic video recordings who underwent treatment at Toho University Hospital between 2016 and 2020. We assessed a novel sign through reviewing thoracoscopic video recordings during the air leakage test after bullectomy, partial lung resection, or other treatment. We defined PDI as an intraoperative difficulty with beginning to inflate the lobes of the lung at an airway pressure of 20 cm H_2_O (Fig. 1; Additional file 1: Video 1). We evaluated as positive PDI sign if inflation time of lung was 10 s or more than and the lobe of lung had delayed inflation in spite of normal inflation of the others. Additionally, we examined PDI sign when inflation time was limited to 30 s or more in patients with PDI as more severe criteria. We determined the inflation time from the start of inflation to the time of adequate inflation. Rapid lung inflation was considered a negative PDI sign and assigned an inflation time of zero. Using bronchoscopy, we confirmed the absence of airway problems with anesthesia and that the intraoperative management of sputum in central airway was adequate. We analyzed the relation between the PDI sign and other clinical factors.

The following parameters were recorded from the medical records: patient sex, age, bulla or bleb formation, past history including asthma, intraoperative air leakage, and an underlying condition known to cause pneumothorax. We classified patients 34 years of age or younger with other underlying diseases as the primary spontaneous pneumothorax group, and divided this group into nonsmokers and smokers according to the criteria of Bintcliffe et al. [4] We classified patients 55 years of age or older as the secondary spontaneous pneumothorax group. We compared the clinical features in patients with pneumothorax with those of a control group of 27 patients who underwent wedge resection of the upper lobe for reasons other than pneumothorax (eg, metastatic tumors, benign lesions).

### Statistical analysis

We used the χ^2^ test to compare binomial factors. Either the t test or the Mann–Whitney U test were used to assess differences for each factor. A *p* value of less than 0.05 was considered statistically significant.

## Results

The patient characteristics are provided in Table 1. Of a total of 111 patients with pneumothorax, 85 were 34 years of age or younger. Of these, 64 were nonsmokers and 21 were smokers. There were 26 patients with 55 years of age or older. In the control group of 27 patients, 20 had metastatic lesions and seven had benign lesions (five with inflammatory changes, one cystic lesion, one hamartoma).

**Table 1:** Characteristics of patients with pneumothorax

Clinical factors	Total	≤ 34 years of age, nonsmokers	≤ 34 years of age, smokers	≥ 55 years of age	Control group
Number of patients (%)	138 (100)	64 (46.4)	21 (15.2)	26 (18.8)	27 (19.6)
Age, mean	38	20	25	66	64
*Sex*					
Male:Female	118:20	58:6	21:0	26:0	13:14
*Laterality*					
Right:Left	71:67	32:32	9:12	16:10	14:13
*Smoking*					
Yes:No	62:76	0:64	21:0	26:0	15:12
*Asthma*					
Yes:No	16:122	9:55	4:17	2:24	1:26
*Bulla or bleb*					
Yes:No	106:32	63:1	20:1	23:3	0:27
*Intraoperative air leakage from bulla or bleb*					
Yes	43	17	8	18	0
*Type of surgery*					
Bullectomy	104	63	21	20	0
Partial resection	28	0	0	1	27
Other	6	1	0	5	0

There was a significant male predominance in patients with pneumothorax compared with the control group (*p* < 0.0001). There were no significant differences between patients with pneumothorax and the control group in either smoking status or the presence of asthma (Table 1; smoking, *p* = 0.3067; asthma, *p* = 0.1964). Of the 111 patients, 106 patients (95.5%) had a bulla or bleb. Of these 106 patients, 40 patients (37·7%) had confirmed intraoperative air leakage from the bulla or bleb; we were not able to confirm the point of air leakage in the remaining 66 patients (62.3%) intraoperatively.

The PDI sign was observed in 78 of the 138 total patients. Of these, 75 patients had the PDI sign in the upper lobe (Table 2). The other three patients were all 55 years of age or older and had a PDI sign observed in the lower lobe.

**Table 2:** Relation between PDI and clinical factors

PDI sign	Total	≤ 34 years of age, nonsmokers	≤ 34 years of age, smokers	≥ 55 years of age	Control group
*PDI sign*					
Positive	78	52	17	5	4
Upper lobe	75	52	17	2	4
Lower lobe	3	0	0	3	0
Negative	60	12	4	21	23
*Sex*					
Male positive (upper)/ negative	66/52	46/12	17/4	2/24	1/12
Female positive (upper)/ negative	9/11	6/0	0/0	0/0	3/11
*Laterality*					
Right positive (upper)/ negative	38/33	27/5	7/2	1/15	3/11
Left positive (upper)/ negative	37/30	25/7	10/2	1/9	1/12
*Asthma*					
Yes positive (upper)/ negative	10/6	7/2	3/1	0/2	0/1
No positive (upper)/ negative	65/57	45/10	14/3	2/22	4/22

PDI: pulmonary delayed inflation

In upper lobe the incidence of the PDI sign in nonsmoking patients 34 years of age or younger was significantly higher than the incidence in patients 55 years of age or older (Table 2; *p* < 0.0001) and patients in the control group (*p* < 0.0001). There was no significant difference between nonsmoking and smoking patients 34 years of age or younger (*p* > 0.9999).

Patients 34 years of age or younger who smoked had a significantly higher incidence of the PDI sign than those 55 years of age or older (Table 2; *p* < 0.0001) and those in the control group (*p* < 0.0001).

There was no significant difference in the incidence of the PDI sign noted between patients 55 years of age or older and patients in the control group. There was also no significant difference noted by sex, the laterality of the operative lung, or the presence of asthma (Table 2).

When inflation time was limited to 30 s or more in patients with PDI, nonsmokers 34 years of age or younger had a significantly higher incidence of PDI than patients 55 years of age or older (Table3; *p* < 0.0001), and patients in the control group (*p* = 0.0003). There was no significant difference between smoking and nonsmoking patients 34 years of age or younger (*p* = 0.8449).

**Table 3:** Relations between PDI lasting 30 s or more with clinical factors

PDI sign	Total	≤ 34 years of age, nonsmokers	≤ 34 years of age, smokers	≥ 55 years of age	Control group
*PDI sign*					
positive: negative	48:90	33:31	12:9	0:26	3:24
*Sex*					
Male:Female (positive/ negative)	43/75:5/15	31/27:2/4	12/9:0/0	0/26:0/0	0/13:3/11
Right:Left (positive/ negative)	24/47:24/43	16/16:17/15	6/3:6/6	0/16:0/10	2/12:1/12
*Asthma*					
Yes:No (positive/ negative)	8/8:40/82	6/3:27/28	2/2:10/7	0/2:0/24	0/1:3/23

PDI: pulmonary delayed inflation

Nonsmokers 34 years of age or younger had a significantly higher incidence of PDI than patients 55 years of age or older (*p* < 0.0001), and patients in the control group (*p* = 0.0003). There was no significant difference between smoking and nonsmoking patients 34 years of age or younger (*p* = 0.8449)

Patients 34 years of age or younger who were smokers had a significantly higher incidence of the PDI sign than patients 55 years of age or older (*p* < 0.0001), and patients in the control group (*p* = 0.0013)

Patients 34 years of age or younger who were smokers had a significantly higher incidence of the PDI sign than patients 55 years of age or older (*p* < 0.0001), and patients in the control group (*p* = 0.0013).

There was no significant difference noted by sex, the laterality of the operative lung, or the presence of asthma (Table 3).

Table 4 shows the relation between inflation time and clinical factors. The inflation time in nonsmoking patients 34 years of age or younger was significantly longer than that in patients 55 years of age or older (*p* < 0.0001) and that noted in the control group (*p* = 0.0059). There was no significant difference between smoking and nonsmoking patients 34 years of age or younger (*p* = 0.3109).

**Table 4:** Inflation time in the upper lobes

PDI sign	Total	≤ 34 years of age, nonsmokers	≤ 34 years of age, smokers	≥ 55 years of age	Control group
Number	138	64	21	26	27
Inflation time, mean, seconds ± SD	30.8 ± 48.7	42.3 ± 47.8	55.6 ± 62.5	1.9 ± 6.7	12.2 ± 43.3

PDI: pulmonary delayed inflation

SD: standard deviation

The inflation time in nonsmoking patients 34 years of age or younger was significantly longer than that in patients 55 years of age or older (*p* < 0.0001) and that noted in the control group (*p* = 0.0059). There was no significant difference between smoking and nonsmoking patients 34 years of age or younger (*p* = 0.3109)

The inflation time in smokers 34 years of age or younger was significantly longer than that noted in patients 55 years of age or older (*p* < 0.0001), and patients in the control group (*p* = 0.0067)

The inflation time in smokers 34 years of age or younger was significantly longer than that noted in patients 55 years of age or older (*p* < 0.0001), and patients in the control group (*p* = 0.0067).

Table 5 shows that there was no significant difference noted in the inflation time in patients with and without asthma.

**Table 5:** Inflation time in the upper lobes in patients with asthma

PDI sign	Total	≤ 34 years of age, nonsmokers	≤ 34 years of age, smokers
Number	85	64	21
Asthma	13	9	4
Asthma Inflation time, mean, seconds ± SD	63.5 ± 79.0	58.1 ± 64.8	75.8 ± 116.0
No asthma Inflation time, mean, seconds ± SD	42.3 ± 45.2	39.7 ± 44.7	50.8 ± 47.2
p value	0.5335	0.2883	0.4870

PDI: pulmonary delayed inflation

SD: standard deviation

## Discussion

The novel PDI sign indicates insufficient lung inflation despite adequate airway pressure. We theorize that it is caused by stenosis of the peripheral airways, revealing the grade of peripheral airway disturbance because we confirmed the absence of airway problems with anesthesia including the absence of sputum in central airway intraoperatively. We conducted a literature search using PubMed and found no previous reports of this finding.

Although secondary spontaneous pneumothorax is caused by COPD in older patients who smoke, the reason for the development of bullas, blebs, and porous visceral pleura is unknown. Although smoking may be related to the development of bullas and blebs in young patients [4], these findings are also observed in young nonsmokers. Some authors report that bullas and blebs may be related to a hereditary predisposition [9], anatomic abnormalities [10, 11], ectomorphic body habitus [6], apical ischemia [12, 13], anorexia nervosa [14], or connective tissue abnormalities [7, 15]. However, none of these have been definitively proven as the cause.

Almost all patients in our study with primary spontaneous pneumothorax had bullas or blebs present. We consider the presence of a bulla or bleb to be the main etiology behind spontaneous pneumothorax, because 40 of our patients (37·7%) with bullas and blebs experienced intraoperative air leakage from those lesions. Pathologic examination of specimens obtained from patients with primary spontaneous pneumothorax reveal bronchiolitis with infiltration of inflammatory cells into the alveolar and interlobular septae, peribronchiolar zones, and the visceral pleura [8]. Therefore, we believe that the PDI sign reveals stenosis of the peripheral airway resulting from bronchitis. When we studied the intraoperative video recordings, we noticed that some patients had this finding predominantly in the upper lobe, while other lobes (middle or lower lobes) were normal. When we consider the etiology of bullas and blebs, it is very important to consider that the PDI sign is mostly seen in young, nonsmoking patients with primary spontaneous pneumothorax, and that it is typically seen in the upper lobe, where bullas and blebs are frequently located. Although there were no differences on frequency of PDI sign or inflation time between patients without smoking and patients with smoking in young patients, we think that it is difficult for young patients to receive irreversible influence due to smoking such as COPD. Because the PDI sign is observed in patients with a primary bulla or bleb but not frequently observed in patients with COPD, the PDI sign may be related to the formation of bullas and blebs in young patients with spontaneous pneumothorax, regardless of smoking status.

Based on our observations of the PDI sign, we propose that bullas and blebs may be caused by high pressure due to air trapping from a check-valve mechanism, which indicates peripheral airway stenosis. If so, bullas and blebs may be prevented by resolving peripheral airway abnormalities in the upper lobes. Further study, including prospective analysis, is needed to determine whether this will prevent spontaneous pneumothorax.

Thoracic surgeons sometimes note abnormalities in intraoperative lung inflation in patients with spontaneous pneumothorax, but these findings are not widely reported. It is possible that surgeons attribute the abnormalities to problems with anesthesia or insufficient intraoperative management of sputum. We have previously failed to report these findings for precisely this reason. Although we reviewed the intraoperative bronchoscopy, we could not observe an association between airway sputum and insufficient lung inflation.

The PDI sign is not related to COPD or to partial lung resection. Nor is it related to patient sex, laterality of the pneumothorax, or asthma. We initially thought that the presence of asthma would be related to the PDI sign, because asthma indicates an abnormality with inflammation of the peripheral airway; however, there was no significant relation between asthma and the PDI sign in our study. We guess that it was influenced by our criteria of PDI sign that the lobe of lung had delayed inflation in spite of normal inflation of the others because bronchial asthma might be usually associated with all lobes. Tanaka et al. noted that only two patients of 67 with secondary spontaneous pneumothorax had confirmed asthma, while 22 patients had emphysema and 21 had tuberculosis [16]. In a study from the United States, only four of 45 patients with pneumothorax and associated pulmonary disease had asthma [17]. However, there are reports relating asthma to pneumothorax [18]. In our study, nine of 64 nonsmoking patients with 34 years or younger had asthma. More studies may be needed to elucidate a potential relation.

There are limitations to our study, including the fact that this is a retrospective study conducted at a single institution. We include comparatively few patients. Despite these limitations, we are able to note significant findings.

## Conclusions

The PDI sign is seen in patients 34 years of age or younger with spontaneous pneumothorax. It may therefore reveal a peripheral bronchial abnormality, and it may be related to the etiology of bleb and bulla formation in young patients. Further study may help to develop effective treatments for pneumothorax in young patients.

## Supplementary information

**Additional file 1**. **Video 1**: Intraoperative findings in patient 1. The left lower lobe is inflated, but the upper lobe is not. A total of 50 s were required to inflate the left upper lobe with an airway pressure of 20 cm H_2_O.

## Data Availability

All data analysed during this study are included in this published article.

## Abbreviations

COPD: Chronic obstructive pulmonary disease
PDI: The pulmonary delayed inflation
